# Infantile Bullous Pemphigoid: Vaccination and SARS-CoV-2 Infection as Triggers

**DOI:** 10.7759/cureus.66303

**Published:** 2024-08-06

**Authors:** Héloise Moens, Louise N Delcambre, Axel De Greef, Marie-Emeline Leboutte, Marie Baeck

**Affiliations:** 1 Dermatology, Cliniques Universitaires Saint-Luc, Brussels, BEL; 2 Pediatrics, Cliniques Universitaires Saint-Luc, Brussels, BEL; 3 Pediatrics, Jolimont Hospital, La Louvière, BEL

**Keywords:** pediatric rare diseases, auto-immune blistering disease, vaccine, sars cov-2, infantile bullous pemphigoid

## Abstract

Bullous pemphigoid (BP) is an acquired auto-immune blistering disease, which is uncommon during childhood. Infantile BP usually has a good prognosis with rare recurrence and the suspected triggers are vaccines or viruses. We report the case of a three-month-old infant girl who presented with BP a week after a SARS-CoV-2 infection and three weeks after the first doses of polio, tetanus, diphtheria, pertussis, Haemophilus influenzae type-b, hepatitis, and pneumococcus vaccinations. Both triggers (infection and vaccination) could be implicated as a slight recurrence was observed after the second doses of vaccines. Rapid clinical resolution was obtained with topical corticosteroids.

## Introduction

Bullous pemphigoid (BP) is a rare disease among children. However, it is the second most common acquired immunobullous disease after linear IgA dermatosis in this life age, with which it shares clinical features. For these reasons, diagnosis is challenging and requires a skin biopsy to be confirmed [1,2]. Scientific evidence is still lacking regarding triggers or treatment management. We report here the case of an infant who presented BP a few days after a documented COVID-19 infection with a slight recurrence after vaccination. The patient responded well to topical treatment.

## Case presentation

A three-month-old girl was admitted for an acral bullous eruption, rapidly spreading to the face, chest, and arms. Three days before the rash, the infant presented with fever and a positive nasal PCR swab for SARS-CoV-2. There was no relevant family history and the infant was otherwise healthy.

**Figure 1:**
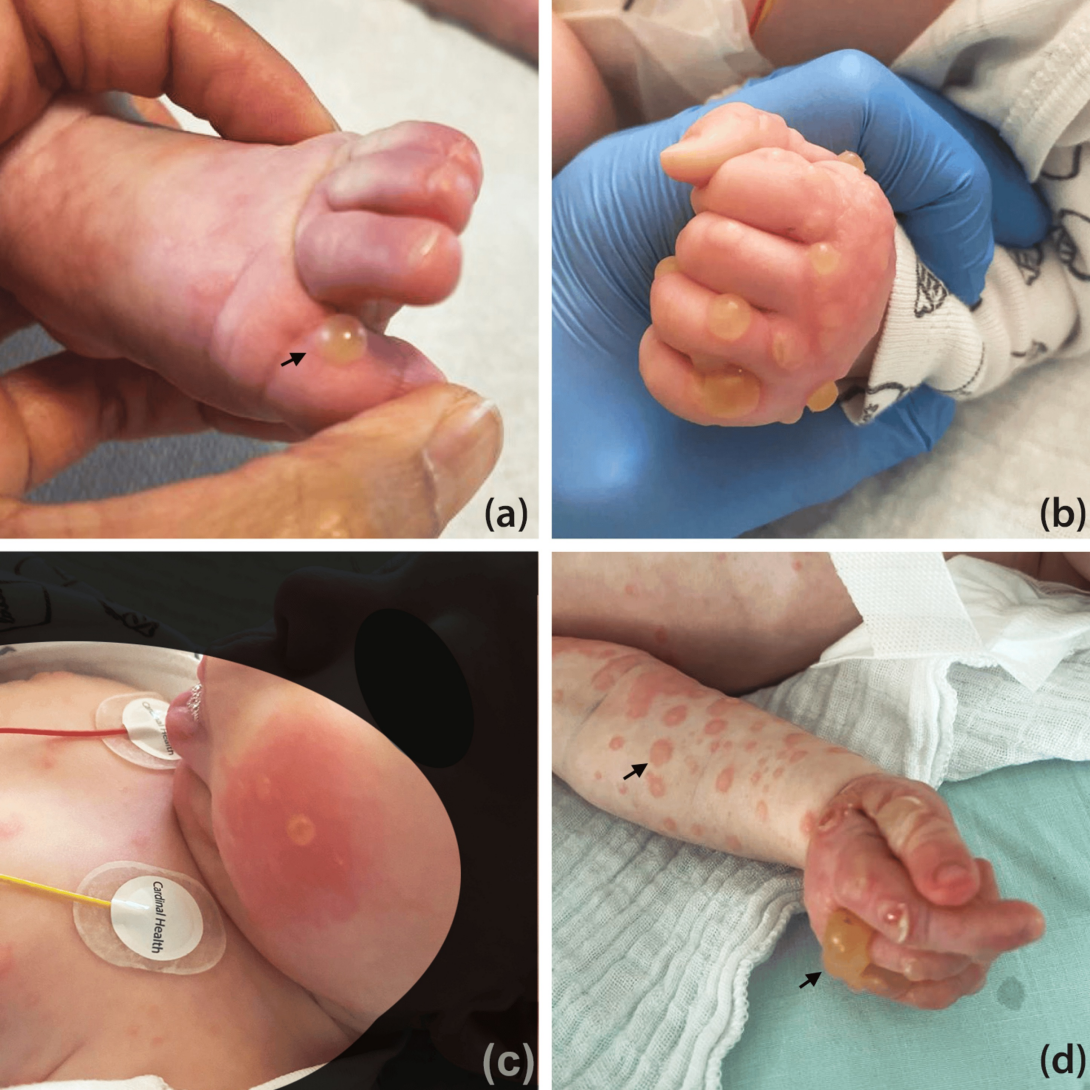
Clinical aspect Tense blisters, erythematous plaques, and erosions on the palms and soles of the feet observed at the start of the eruption (A, B) then extension on the face (C). Circled-like lesions on the arms and the trunk (D).

Paracetamol was administered for the fever and no other medication was recorded. Immunization was started 24 days before the eruption with the first doses of polio, tetanus, diphtheria, pertussis, Haemophilus influenzae type-b, hepatitis, and pneumococcus vaccines. Physical exam revealed vesicles and blisters with erythema and edema on the hands and feet (Figures 1A, 1B). On the trunk and limbs, erythematous macules with an annular appearance were observed (Figures 1C, 1D). The mucosa was not involved. Nikolsky's sign was negative.

**Figure 2:**
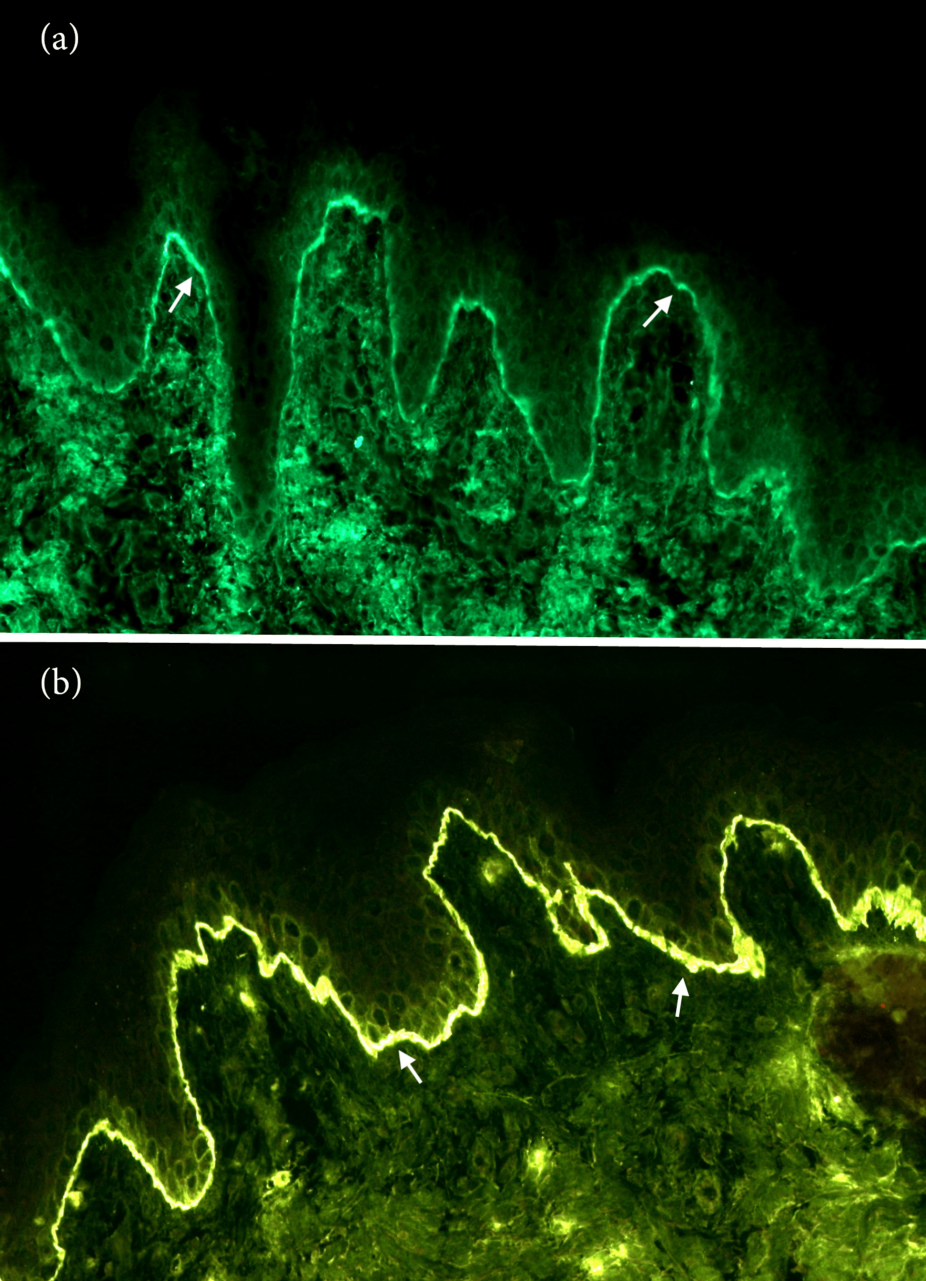
Immunohistochemistry Direct immunofluorescence (DIF) examination of a perilesional skin biopsy. IgG antibodies (A) and C3 linear deposits (B) detected along the dermo-epidermal junction.

Laboratory findings showed moderate eosinophilia (1.090/nL, normal value 0.04-0.58/nL), with no other significant abnormalities. Microbiological analyses were negative. Histopathological examination of a skin biopsy showed an inflammatory dermal infiltrate with numerous eosinophilic polymorphonuclear cells with margination along the dermo-epidermal junction. Direct immunofluorescence revealed IgG antibodies and C3 linear deposits along the dermo-epidermal junction (Figures 2A, 2B). Enzyme-linked immunosorbent assay (ELISA) testing with recombinant BP180 was positive (228.33 U /ml), while it was negative for the recombinant BP230. Based on the clinical features, and histopathological and immunologic findings, the diagnosis of BP was made.

Treatment with topical corticosteroids (mometasone furoate 0,1% - in a thin layer on the lesions once a day) was initiated and led to complete resolution of the lesions within two weeks. Treatment was stopped after a few days of complete resolution. Despite delaying the second dose of vaccines by 10 days, a slight recurrence of the lesions was observed. However, topical corticosteroids, at the same dosage, once again allowed for a rapid improvement. There was no recurrence of the lesions at the third vaccination.

## Discussion

BP is rare during infancy, with two peaks of incidence: in the first year of life and around eight years old [1]. It is the second most common acquired immunobullous disease in children after linear IgA dermatosis [2]. The bullous lesions and the annular erythematous involvement on the limbs and trunk led us to consider IgA dermatosis as the primary differential diagnosis, although the initial acral involvement could have suggested BP. However, an eosinophilic rather than a neutrophilic infiltrate, along with linear deposits of IgG and C3 complements, confirmed the diagnosis of BP. This emphasizes the importance of skin biopsy in the differential diagnosis of bullous lesions in children. Positive autoantibodies against BP180 antigen and negative anti-BP230 are common in the infantile form of BP as in the present case [3,4].

The exact etiology and the triggering factors of infantile BP are not yet known. Research supports the involvement of genetic and environmental factors such as viral infection or immunization [5]. There are several reports related to vaccine administration [6]. The mean time between vaccination and BP was 7.5 days (range: 5 hours to 3 weeks) [7]. In the case of vaccine-induced dermatosis, the hyperinflammation state produced seemed to be initiated by molecular mimicry and immune cross-reaction [8]. However, the causality link is difficult to prove. In most cases, vaccination was pursued with only a few relapses. The first potential trigger in the case herein reported was the proven SARS-CoV-2 infection a few days before the first lesion. In fact, some viruses may induce a nonspecific immune activation and via similarities between viral peptides and self-peptides of skin membrane, leading to an immune response against self-antigens [9]. These autoantibodies react against epidermic basal membrane antigens (BP-180 or BP-230, a component of hemidesmosomes) and cause tissue damage. Potential triggering by COVID-19 has already been reported [10].

Infantile BP has generally a good prognosis and resolves rapidly after treatment. Topical or systemic corticosteroids remain the first-line treatment. If necessary, immunosuppressive agents can be added such as dapsone, mycophenolate mofetil or cyclosporine. Other treatments such as sulfapyridine, erythromycin ethulsuccinat, intra-venous immunoglobulins, rituximab, and omalizumab have been proposed in refractory BP [1,3,11].

## Conclusions

BP is a rare disease among children but should be considered in the case of childhood bullous eruption with predominant acral involvement. This case report is original in that two triggering factors are potentially involved or even associated. To our knowledge, few cases have been reported following infection with SARS-CoV-2. Monotherapy with topical corticosteroids was effective, even after relapse, thus avoiding potential side effects of oral steroids. Further studies are needed to improve treatment guidelines and better assess the triggers of this disease.

## References

[1] Waisbourd-Zinman O, Ben-Amitai D, Cohen AD (2008). Bullous pemphigoid in infancy: clinical and epidemiologic characteristics. J Am Acad Dermatol.

[2] Howell S, Enginli A, Strowd L, Ahn C (2021). A case report of bullous pemphigoid in infancy. J Am Acad Dermatol.

[3] Schwieger-Briel A, Moellmann C, Mattulat B (2014). Bullous pemphigoid in infants: characteristics, diagnosis and treatment. Orphanet J Rare Dis.

[4] Saad S, Ghariani Fetoui N, Mani O (2023). Pemphigoide bulleuse de l’enfant. Annales de Dermatologie et de Vénérologie.

[5] Lo Schiavo A, Ruocco E, Brancaccio G, Caccavale S, Ruocco V, Wolf R (2013). Bullous pemphigoid: etiology, pathogenesis, and inducing factors: facts and controversies. Clin Dermatol.

[6] Yang M, Wu H, Zhao M, Chang C, Lu Q (2019). The pathogenesis of bullous skin diseases. J Transl Autoimmun.

[7] Neri I, Greco A, Bassi A (2016). Bullous pemphigoid in infant post vaccination: myth or reality?. Int J Immunopathol Pharmacol.

[8] Karampinis E, Papadopoulou MM, Chaidaki K (2024). Plaque psoriasis exacerbation and COVID-19 vaccination: assessing the characteristics of the flare and the exposome parameters. Vaccines (Basel).

[9] Baum H, Butler P, Davies H, Sternberg MJE, Burroughs AK (1993). Autoimmune disease and molecular mimicry: an hypothesis. Trends Biochem Sci.

[10] Rosińska-Więckowicz A, Jałowska M, Bowszyc-Dmochowska M, Dmochowski M (2021). Case report: infantile bullous pemphigoid: triggering by COVID-19 is speculative. Front Med (Lausanne).

[11] Tekin B, Yücelten AD (2015). Infantile bullous pemphigoid treated using intravenous immunoglobulin: case report and review of the literature. Pediatr Dermatol.

